# Pyruvate kinase 2 from *Synechocystis* sp. PCC 6803 increased substrate affinity via glucose-6-phosphate and ribose-5-phosphate for phosphoenolpyruvate consumption

**DOI:** 10.1007/s11103-023-01401-0

**Published:** 2024-05-17

**Authors:** Masahiro Karikomi, Noriaki Katayama, Takashi Osanai

**Affiliations:** School of Agriculture, Meiji University, 1-1-1, Higashimita, Tama-Ku, Kawasaki, Kanagawa 214-8571 Japan

**Keywords:** Glycolysis, Microalgae, Pyruvate kinase, *Synechocystis* sp. PCC 6803

## Abstract

Pyruvate kinase (Pyk*,* EC 2.7.1.40) is a glycolytic enzyme that generates pyruvate and adenosine triphosphate (ATP) from phosphoenolpyruvate (PEP) and adenosine diphosphate (ADP), respectively. Pyk couples pyruvate and tricarboxylic acid metabolisms. *Synechocystis* sp. PCC 6803 possesses two pyk genes (encoded *pyk1*, sll0587 and *pyk2*, sll1275). A previous study suggested that *pyk2* and not *pyk1* is essential for cell viability; however, its biochemical analysis is yet to be performed. Herein, we biochemically analyzed *Synechocystis* Pyk2 (hereafter, *Sy*Pyk2). The optimum pH and temperature of *Sy*Pyk2 were 7.0 and 55 °C, respectively, and the *K*_m_ values for PEP and ADP under optimal conditions were 1.5 and 0.053 mM, respectively. *Sy*Pyk2 is activated in the presence of glucose-6-phosphate (G6P) and ribose-5-phosphate (R5P); however, it remains unaltered in the presence of adenosine monophosphate (AMP) or fructose-1,6-bisphosphate. These results indicate that *Sy*Pyk2 is classified as PykA type rather than PykF, stimulated by sugar monophosphates, such as G6P and R5P, but not by AMP. *Sy*Pyk2, considering substrate affinity and effectors, can play pivotal roles in sugar catabolism under nonphotosynthetic conditions.

**Supplementary Information:**

The online version contains supplementary material available at 10.1007/s11103-023-01401-0.

## Key message

Glucose-6-phosphate and ribose-5-phosphate increased *Synechocystis* Pyk2 affinity for PEP, possibly contributing to PEP consumption under nonphotosynthetic conditions.

**Supplementary Information:**

The online version contains supplementary material available at 10.1007/s11103-023-01401-0.

## Introduction

Cyanobacteria can utilize CO_2_ via photosynthesis to synthesize value-added metabolites for a low-carbon society (Hidese et al. 2020; Angermayr et al. 2014; Hasunuma et al. 2018). *Synechocystis* sp. PCC 6803 (hereafter, *Synechocystis*) is a unicellular, non-nitrogen fixing model cyanobacterium that is utilized for bioproduction (Ruffing 2011; Wang et al. 2012; Yu et al. 2013). *Synechocystis* produces carboxylic acids, such as D-lactate, and polyhydroxy-3-butyrate (PHB), as food additives and bioplastics; these two metabolites are derived from pyruvate (Osanai et al. 2015; Hidese et al. 2020; Ito et al. 2017; Carpine et al. 2017).

**Fig. 1:**
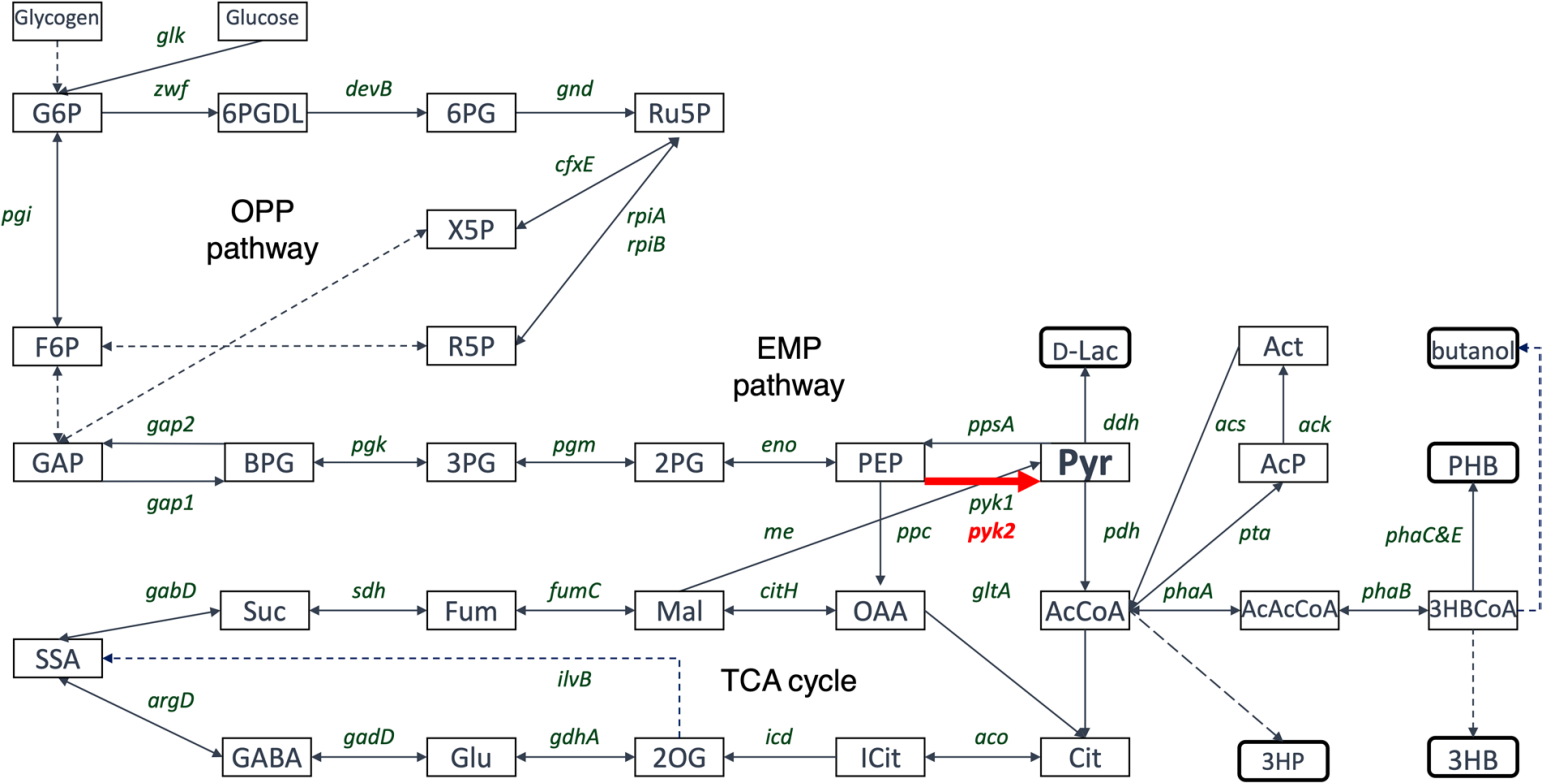
Pathway map of *Synechocystis* sp. PCC 6803 (*Synechocystis*). The metabolic maps of the Embden–Meyerhof–Parnas (EMP) pathway/gluconeogenesis, oxidative pentose phosphate (OPP) pathway, pyruvate metabolism, and tricarboxylic acid (TCA) cycle. The gene names encoding metabolic enzymes in *Synechocystis* were obtained from the Kyoto Encyclopedia of Genes and Genomes database. The rounded rectangle indicated value-added metabolites from *Synechocystis*

Various groups have widely studied primary carbon metabolism in *Synechocystis*, including metabolic regulation, pathway identification, and metabolic enzyme biochemistry (Fig. 1). *Synechocystis* has several glucose catabolic routes, such as the Embden–Meyerhof–Parnas (EMP) and oxidative pentose phosphate (OPP) pathways (You et al. 2014). *Synechocystis* has a unique tricarboxylic acid (TCA) cycle lacking 2-oxoglutarate dehydrogenase and possessing alternative pathways, such as a γ-butyric amino acid (GABA) shunt (Zhang and Bryant 2011; Xiong et al. 2014). The properties of the enzymes of the *Synechocystis* TCA cycle have been studied, including those of citrate synthase (CS encoded by *gltA*, sll0401) (Ito et al. 2019), aconitase (Aco encoded by *acnB*, slr0665) (Nishii et al. 2021; de Alvarenga et al. 2020), isocitrate dehydrogenase (IDH encoded by *icd*, slr1289) (Muro-Pastor and Florencio 1992), malate dehydrogenase (MDH encoded by *citH*, sll0891) (Takeya et al. 2018), malic enzyme (ME encoded by *me*, sll0721) (Katayama et al. 2022), and succinate dehydrogenase (SDH encoded by *sdh*, sll0823 and sll1625) (Cooley and Vermaas 2001). Two enzymes, phosphoenolpyruvate carboxylase (PEPC) and pyruvate kinase (Pyk), catalyze specific reactions that provide carbon sources to the TCA cycle. *Synechocystis* possesses one PEPC encoded by *ppc* (sll0920) and two Pyks encoded by *pyk1* (sll0587) and *pyk2* (sll1275) (Kaneko et al. 1996). *Synechocystis* PEPC exhibits a unique allosteric regulation uninhibited by several metabolic effectors, such as malate, aspartate, and fumarate (Takeya et al. 2017; Scholl et al. 2020). Pyk enzymatic activity from *Synechocystis* cell cultures has been measured and reported to be higher under nonphotosynthetic conditions than those under photoautotrophic and mixotrophic conditions (Knowles and Plaxton 2003).

The phylogenetic analysis of the Pyks of *Synechocystis* revealed bacterial Pyk and an evolutionary distance between the two isoforms of Pyks, Pyk1 (hereafter, *Sy*Pyk1) and Pyk2, in cyanobacteria (Haghighi 2021). Bacterial Pyk is classified as PykA and PykF, and sugar monophosphates, such as AMP, G6P, and R5P, stimulate PykA. PykF is activated by sugar diphosphates, such as FBP, in *Escherichia coli* (Kornberg and Malcovati 1973; Waygood et al. 1975, 1976). *Sy*Pyk2 is classified into *pykF*, and *pyk2* knockout causes severe growth defects (Yao et al. 2020). In a previous study, the biochemical analysis of the Pyk of another cyanobacterium, *Synechococcus* sp. PCC 6301 (hereafter, *Synechococcus* Pyk), was reported to be homologous to that of *Synechocystis pyk2*, suggesting that ATP and TCA metabolism inhibited *Synechococcus* Pyk, thus indicating its roles under dark conditions (Knowles et al. 2001). *pyk2* expression increases in the wild-type strain in the presence of glucose (Kaniya et al. 2013). *pyk2* expression patterns remain unchanged during the day/night cycle (Saha et al. 2016). These reports indicate that *Sy*Pyk2 is essential during photosynthetic and nonphotoautotrophic conditions in *Synechocystis*. This study revealed the regulatory properties of *Sy*Pyk2 via biochemical analysis and demonstrated that sugar phosphates activated *Sy*Pyk2 activity.

## Materials and methods

### Construction of cloning vector for recombinant *Sy*Pyk2

The amino acid sequence of Pyk2 (sll1275) polypeptide was obtained from the Kyoto Encyclopedia of Genes and Genomes database (https://www.genome.jp/kegg/) and synthesized by Eurofins Genomics Japan (Tokyo, Japan). The synthesized fragment was inserted within the BamHI–XhoI site of the vector pGEX6P-1 (GE Healthcare Japan, Tokyo, Japan). The cloned expression vector was transformed into competent *E*. *coli* BL21 cells (Takara Bio, Shiga, Japan) and cultured in 6 mL of Luria–Bertani medium at 30 °C with shaking at 150 rpm. Recombinant *E. coli* BL21 cells from 1.2-L culture were suspended in 40 mL of phosphate-buffered saline/Tween (PBST) (1.37 M NaCl, 27 mM KCl, 81 mM Na_2_HPO_4_・12H_2_O, 14.7 mM KH_2_PO_4_, and 0.05% Tween 20) and sonicated (model VC-750; EYELA, Tokyo, Japan). This procedure was repeated 10 times at 20% intensity for 20 s. The lysed cells were centrifuged twice at 12,500 rpm for 15 min at 4 °C. The supernatant was transferred into a 50 mL tube, and 2 mL of Glutathione Sepharose 4 B resin (Cytiva Japan, Tokyo, Japan) was added. The tubes were shaken gently for 60 min on ice. The mixture was centrifuged at 8,000 rpm for 2 min at 4 °C to remove the supernatant. The resin was transferred to a 15 mL tube and washed using PBST. After washing, the recombinant protein was eluted five times using 650 μL of glutathione-S-transferase (GST) elution buffer (50 mM Tris–HCl, pH 9.6-, and 10-mM reduced glutathione) and incubated in Vivaspin 500 MWCO 50,000 device (Sartorius, Göttingen, Germany) for protein concentration. The protein concentrations were determined using Pierce BCA Protein Assay Kit (Thermo Fisher Scientific, Rockford, IL, USA). The solution was transferred to a 1.5 mL tube, and 40 units of PreScission Protease (equivalent to the purified recombinant protein) (Cytiva) were added and allowed to stand at 4 °C for 16 h to remove GST-tag from the recombinant proteins. Approximately 750 µL of Glutathione Sepharose 4B resin was added, rotated for 1 h at room temperature to remove the cleaved tag from the solution, at 11,000 rpm for 4 min at 4 °C, and the supernatant was collected. Sodium dodecyl sulfate–polyacrylamide gel electrophoresis was performed to confirm protein purification, and gels were stained using Quick Blue reagent (BioDynamics Inc. Tokyo, Japan).

### Enzyme assay

All solutions were prepared using Milli-Q water; the *Sy*Pyk2 reaction was coupled with lactate dehydrogenase (LDH) from a pig heart (Wako Chemicals, Osaka, Japan) reaction and measured at 30 °C or 55 °C by monitoring NADH oxidation at 340 nm in a final volume of 1 mL. The experiments were performed using 1.88 pmol of the recombinant *Sy*Pyk2 proteins. We measured *Sy*Pyk2 activity at an intracellular condition of 30  °C and pH 7.8 (Inoue et al. 2001; Nakamura et al. 2021). Unless otherwise indicated, the assay conditions for *Sy*Pyk2 were 100 mM Tris–HCl (pH 7.8) or 100 mM MES-NaOH (pH 7.0), 15 mM MgCl_2_ or 5 mM MnCl_2_, 100 mM KCl, 0.2 mM NADH, 2 mM ADP-2Na, 15 units/mL desalted LDH from pig heart, and 5 mM PEP-Na. All measurements were performed using 15 mM Mg^2+^ except where indicated. One unit of *Sy*Pyk2 activity was defined as the oxidation of 1 μmol NADH per minute produced. Each effector was added 0.1 mM each: glucose-6-phosphate-2Na (G6P); fructose-6-phosphate-2Na (F6P); fructose-1, 6-bisphosphate-3Na (FBP); ribose-5-phosphate-2Na (R5P); 6-phospho-D-gluconate (6PG); adenosine monophosphate-Na (AMP); adenosine diphosphate-2Na (ADP); adenosine triphosphate-2Na (ATP); citrate-3Na; 2-oxoglutarete (2OG); succinate-2Na; fumarate; malate-Na. The results were plotted as a graph of the reaction rate to substrate and coenzyme concentration. *K*_m_ and *V*_max_ values were calculated by curve fitting using Kaleida Graph ver. 4.5 software and *k*_cat_ were calculated from the *V*_max_.

### Statistical analyses

Paired two-tailed Student’s *t*-tests were performed to calculate the *P*-values using Microsoft Excel for Windows (Redmond, WA, USA). All experiments were conducted independently in triplicate.

## Results

### Purification of *Sy*Pyk2 and determination of optimal temperature and pH

**Fig. 2:**
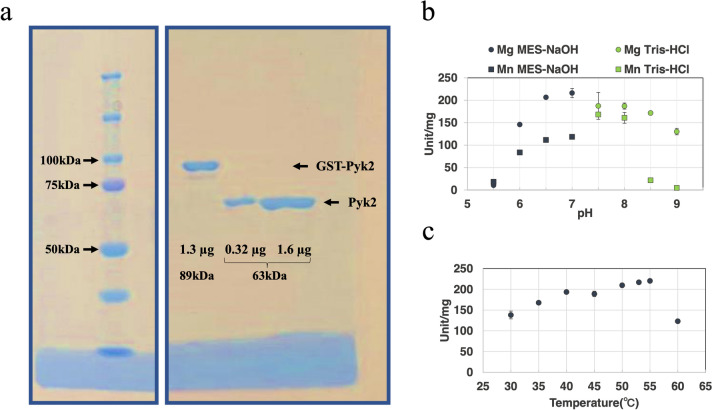
Sodium dodecyl sulfate–polyacrylamide gel electrophoresis (SDS-PAGE) and optimal pH and temperature for *Synechocystis* pyruvate kinase 2 (*Sy*Pyk2). **a** Purified GST-tagged *Sy*Pyk2 (89 kDa) and untagged *Sy*Pyk2 (63 kDa) proteins. GST-Pyk2 indicated GST-tagged *Sy*Pyk2, and Pyk2 indicated untagged *Sy*Pyk2. The gel was prepared using 8% (w/v) acrylamide and stained with Quick Blue G250. Optimum pH and temperature for *Sy*Pyk2. **b** Effects of the pH on *Sy*Pyk2 activity. The circle and square represented Mg^2+^ and Mn^2+^, respectively. Blue and green represented the buffer MES-NaOH and Tris–HCl, respectively. The concentrations of phosphoenolpyruvate (PEP), adenosine diphosphate (ADP), and KCl were fixed at 5.0, 2.0, and 100 mM, respectively. The experiments of Mn^2+^ measured at pH 8.5 and 9.0 Tri-HCl was precipitated. The mean ± SD values were calculated from three independent experiments. **c** The effects of temperature on *Sy*Pyk2 activity. This experiment was measured in MES-NaOH buffer pH 7.0, and 15 mM Mg^2+^ of the cofactor was used. PEP, ADP, and KCl concentrations were fixed at 5.0, 2.0, and 100 mM, respectively. The mean ± SD values were calculated from three independent experiments

We expressed GST-tagged *Sy*Pyk2 proteins in *E*. *coli* BL21 and purified them using affinity chromatography (Fig. 2a). *Sy*Pyk2 activity for PEP was the highest in MES-NaOH buffer at pH 7.0 and temperature 55 °C (Fig. 2b and c). The experiments measured at pH 8.5 and 9.0 Tri-HCl using Mn^2+^ was precipitated (Fig. 2b). Following this, *Sy*Pyk2 activities for PEP were measured under optimal conditions (55 °C and pH 7.0) or intracellular conditions (30 °C and pH 7.8).

### Dependence *Sy*Pyk2 cations for catalytic activity

**Fig. 3:**
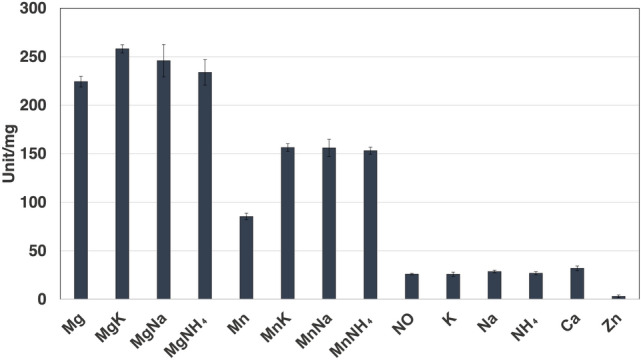
Effects of cofactor monovalent and divalent cations for *Synechocystis* pyruvate kinase 2 (*Sy*Pyk2) activity. The monovalent and divalent cations were fixed at 100 and 5 mM, respectively, except for MgCl_2_ and ZnCl_2_ fixed at 15 and 0.5 mM. The experiment was performed using 100 mM MES-NaOH buffer (pH 7.0) at 55 °C. The concentrations of PEP and ADP were fixed at 5.0 and 2.0 mM, respectively. The mean ± SD values were calculated from three independent experiments. K, KCl; Na, NaCl_2_; NH_4_, NH_4_Cl; Mg, MgCl_2_·6H_2_O; Mn, MnCl_2_·4H_2_O; Ca, CaCl_2_; Zn, ZnSO_4_·7H_2_O

**Fig. 4:**
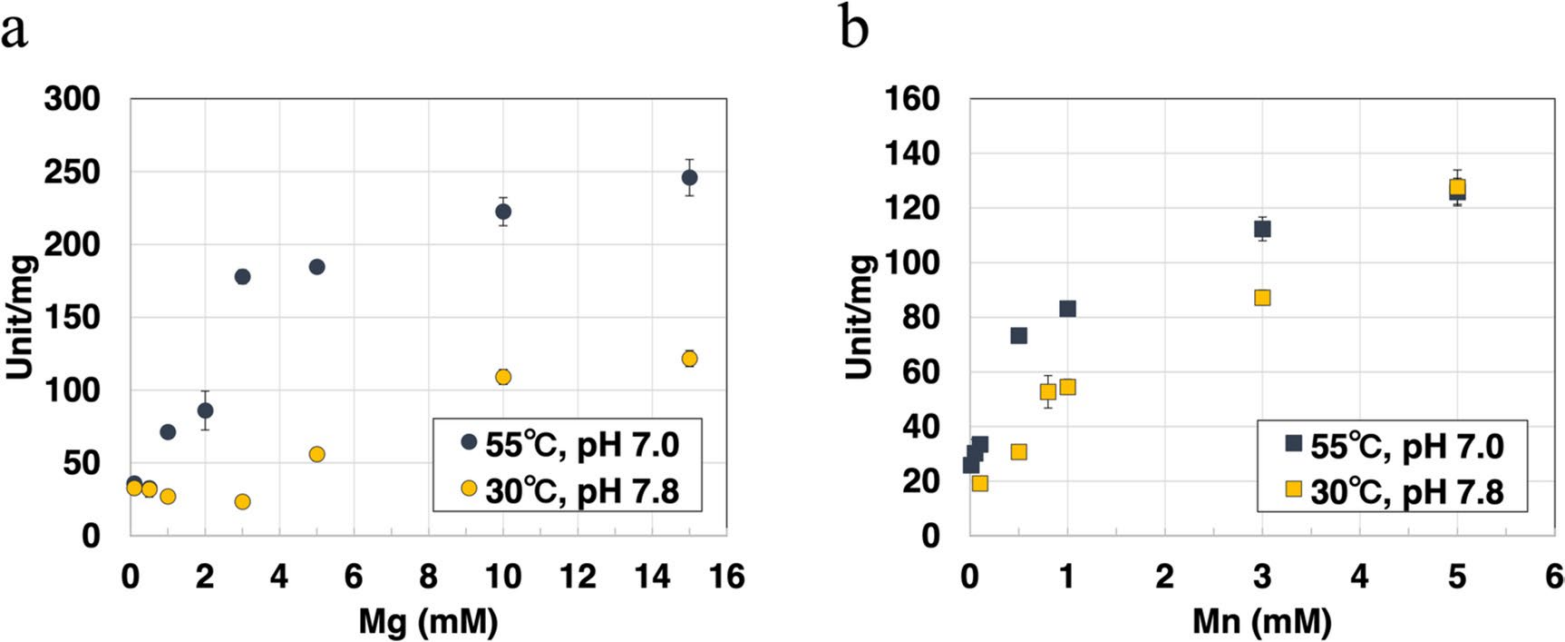
*Synechocystis* pyruvate kinase 2 (*Sy*Pyk2) activity at different concentrations of MgCl_2_ (**a**) and MnCl_2_ (**b**). These experiments were performed under optimum conditions at 55 °C and pH 7.0 in MES-NaOH buffer (blue) or intracellular conditions at 30 °C and pH 7.8 in Tris–HCl buffer (yellow). These experiments fixed the phosphoenolpyruvate (PEP), adenosine diphosphate (ADP), and KCl concentrations at 5.0, 2.0, and 100 mM, respectively. The mean ± SD values were calculated from three independent experiments

Similar to the other bacterial Pyks (Waygood and Sanwal 1974; Kapoor and Venkitasubramanian 1983; Wu and Turpin 1992; Snášel and Pichová 2019), *Sy*Pyk2 activity was dependent on divalent cations such as Mg^2+^ or Mn^2+^, and the *V*_max_ (maximum reaction velocity) of *Sy*Pyk2 activity in the presence of Mn^2+^ was half of that in the presence of Mg^2+^ (Figs. 3, 4a, and b). The activity of *Sy*Pyk2 was higher in the presence of divalent cations than in the presence of monovalent cations, and its activation by monovalent cations was not K^+^-specific (Fig. 3). We determined the kinetic parameters of *Sy*Pyk2 with respect to its dependence on Mg^2+^ and Mn^2+^. Under optimal conditions, the *K*_m_ (half-saturation concentration) value of *Sy*Pyk2 for Mg^2+^ and Mn^2+^ dependence was 3.54 ± 0.61 and 0.296 ± 0.02 mM, respectively (Fig. 4a and b). Under intracellular conditions, the *K*_m_ value of *Sy*Pyk2 for Mg^2+^ and Mn^2+^ dependence was 6.70 ± 0.26 and 2.18 ± 0.51 mM, respectively (Fig. 4a and b). The *K*_m_ value of *Synechococcus* Pyk and *Synechocystis* PEPC for Mg^2+^ dependence were 2.9 and 4.27 ± 0.46 mM, respectively (Knowles et al. 2001; Scholl et al. 2020). Thus, we defined that the optimum conditions for *Sy*Pyk2 were 15 mM MgCl_2_ and 5 mM MnCl_2_.

### Determination of kinetic parameters of *Sy*Pyk2

**Fig. 5:**
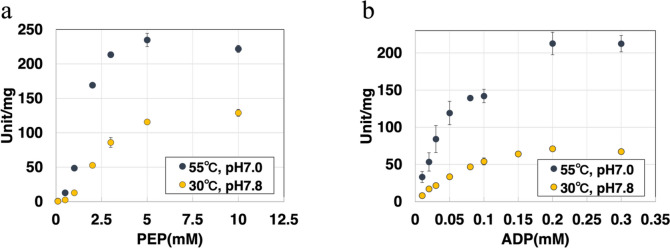
Saturation curves of *Synechocystis *pyruvate kinase 2 (*Sy*Pyk2) for phosphoenolpyruvate (PEP) and adenosine diphosphate (ADP). **a** The saturation curves of *Sy*Pyk2 for PEP. These measurements were performed in an optimum condition at 55 °C and pH 7.0 in MES-NaOH buffer (blue) or an intracellular condition of 30 °C and pH 7.8 in Tris–HCl (yellow). The ADP concentration was 2.0 mM. The mean ± SD values were calculated from three independent experiments. **b** The saturation curves of *Sy*Pyk2 for ADP. These measurements were performed in an optimum condition at 55 °C and pH 7.0 in MES-NaOH buffer (blue) or an intracellular condition of 30 °C and pH 7.8 in Tris–HCl (yellow). The PEP concentration was 5 mM. The concentrations of KCl and MgCl_2_ were 100 and 15 mM, respectively. The mean ± SD values were calculated from three independent experiments

We measured the kinetic parameters of *Sy*Pyk2 for PEP and ADP under optimal conditions. The saturation curves of *Sy*Pyk2 for PEP displayed a sigmoidal curve with *V*_max_ and *K*_m_ of 241 ± 10.5 unit/mg and 1.53 ± 0.07 mM, respectively, and a Hill coefficient of 3.10 ± 0.11, indicating positive homotropic cooperativity (Fig. 5a and Table 1). The saturation curves of *Sy*Pyk2 for ADP followed a hyperbolic (Michaelis–Menten) curve with *V*_max_ and *K*_m_ of 239 ± 6 unit/mg and 0.0527 ± 0.0075 mM, respectively (Fig. 5b and Table 1). The saturation curves of *Sy*Pyk2 for PEP and ADP under intracellular conditions were determined. The saturation curves of *Sy*Pyk2 for PEP showed a sigmoidal curve with *V*_max_ and *K*_m_ of 119 ± 7 unit/mg and 2.54 ± 0.12 mM, respectively, and a Hill coefficient of 2.60 ± 0.18, suggesting positive homotropic cooperativity (Fig. 5a and Table 1). The saturation curves of *Sy*Pyk2 for ADP exhibited a hyperbolic curve with *V*_max_ and *K*_m_ of 80.3 ± 5.3 Unit/mg and 0.0602 ± 0.0081 mM, respectively (Fig. 5b and Table 1). Similar to *Synechococcus* Pyk and other bacterial Pyks, the saturation curves of *Sy*Pyk2 for PEP showed sigmoidal curves (Knowles et al. 2001; Jetten et al. 1994; Abdelhamid et al. 2019; 2021), and for ADP, hyperbolic curves (Abdelhamid et al. 2019; 2021). Additionally, we measured the activity of *Sy*Pyk2 at 30 °C and pH 7.0; the conditions were optimal for *Synechocystis* PEPC (Takeya et al. 2017) and competed with *Sy*Pyk2 for PEP consumption. The saturation curves of *Sy*Pyk2 for PEP displayed a sigmoidal curve with *V*_max_ and *K*_m_ of 132 ± 5 unit/mg and 2.36 ± 0.2 mM, respectively, and a Hill coefficient of 2.61 ± 0.18, indicating positive homotropic cooperativity and intracellular conditions (Supplemental Fig. 1a and Table 2). The saturation curves of ADP followed a hyperbolic curve with *V*_max_ and *K*_m_ of 123 ± 9 unit/mg and 0.111 ± 0.017 mM, respectively (Supplemental Fig. 1b and Table 2). In these conditions, the *K*_m_ value of *Sy*Pyk2 for PEP was approximately 30-, 40-, or 20-fold higher than that of *Sy*Pyk2 for ADP under optimum, intracellular, and optimum for *Synechocystis* PEPC conditions, respectively (Tables 1 and 2). The *K*_m_ value of *Sy*Pyk2 for ADP was half of that of *Synechococcus* Pyk, and the *K*_m_ value of *Sy*Pyk2 for PEP was 5-fold higher than that of *Synechococcus* Pyk (Knowles et al. 2001). Compared with the *K*_m_ value of *Synechocystis* PEPC (0.34 mM: Takeya et al. 2017, 0.88 mM: Scholl et al. 2020) for PEP, *Sy*Pyk2 required more than 2-fold PEP (Tables 1 and 2).

**Table 1:** Kinetic parameters of pyruvate kinase 2 under optimal and intracellular conditions

	*V*_max_ (Unit/mg)	*K*_m_ (mM)	*k*_cat_ (s^−1^)	*k*_cat_/*K*_m_ (s^−1^ mM^−1^)	*n*_*H*_
PEP (55 °C, pH 7.0)	241 ± 10.5	1.53 ± 0.07	253 ± 11	166 ± 3	3.10 ± 0.11
ADP (55 °C, pH 7.0)	238 ± 5	0.0527 ± 0.0076	251 ± 6	4837 ± 733	1.25 ± 0.11
PEP	119 ± 7	2.54 ± 0.118	126 ± 8	49.4 ± 1.2	2.64 ± 0.18
ADP	80.3 ± 5.3	0.0602 ± 0.0081	84.5 ± 5.6	1411 ± 100	1.37 ± 0.24
PEP + ATP	107 ± 5	2.74 ± 0.09	114 ± 5	41.2 ± 0.4	3.06 ± 0.65
PEP + G6P	122 ± 5	0.607 ± 0.009	128 ± 5	212 ± 9	2.00 ± 0.10
PEP + G6P & ATP	130 ± 30	0.62 ± 0.22	137 ± 31	227 ± 25	1.77 ± 0.36
PEP + R5P	125 ± 18	0.548 ± 0.132	131 ± 19	243 ± 29	1.41 ± 0.15
PEP + R5P & ATP	83.0 ± 7.2	0.613 ± 0.100	91.9 ± 7.6	151 ± 10	1.66 ± 0.22

The kinetic parameters of the activity of *Synechocystis* pyruvate kinase 2 (*Sy*Pyk2) in the presence of activators (glucose-6-phosphate, G6P and ribose-5-phosphate, R5P) or inhibitor (adenosine triphosphate, ATP) and coexisting metabolites. All experiments were performed under intracellular conditions of 30 °C and pH 7.8 in Tris–HCl buffer, except for those marked as 55 °C and pH 7.0 in MES-NaOH buffer. Each kinetic parameter is explained as follows:* V*_max_ (maximum reaction velocity); *K*_m_, half-saturation concentration; *k*_cat_, turnover number; *k*_cat_/*K*_m_, catalytic efficiency; *n*_H_, Hill coefficient. Mean ± SD values were calculated from three independent experiments

**Table 2:** Kinetic parameters of *Synechocystis* pyruvate kinase 2 and PEPC under optimal conditions for *Synechocystis* PEPC

	*V*_max_ (Unit/mg)	*K*_m_ (mM)	*k*_cat_ (s^−1^)	*k*_cat_/*K*_m_ (s^−1^ mM^−1^)	*n*_*H*_
PEP (30 °C, pH 7.0)	132 ± 5	2.36 ± 0.20	139 ± 5	59.1 ± 3.1	2.61 ± 0.17
ADP (30 °C, pH 7.0)	123 ± 9	0.111 ± 0.017	130 ± 9	1179 ± 90	0.76 ± 0.04
*Synechocystis* PEPC (Takeya et al. 2017) (30 °C, pH7.0)	1.74	0.34	na	na	na
*Synechocystis* PEPC (Scholl et al. 2020) (28 °C, pH 8.0)	24.2 ± 2.30	0.88 ± 0.15	48.71 ± 4.63	na	na

This table shows the kinetic parameters of the activity of *Synechocystis* pyruvate kinase 2 (*Sy*Pyk2) under optimal conditions for *Synechocystis* phosphoenolpyruvate carboxylase (PEPC) at 30 °C and pH 7.0 (Takeya et al. 2017). The experiments *Sy*Pyk2 were performed at 30 °C and pH 7.0 in MES-NaOH buffer. Each kinetic parameter is explained as follows: *V*_max_ (maximum reaction velocity); *K*_m_, half-saturation concentration; *k*_cat_, turnover number; *k*_cat_/*K*_m_, catalytic efficiency; *n*_H_, Hill coefficient; na, data not available. The mean ± SD values were calculated from three independent experiments

### Activation and inhibition of *Sy*Pyk2 by sugar phosphates and organic acids

**Fig. 6:**
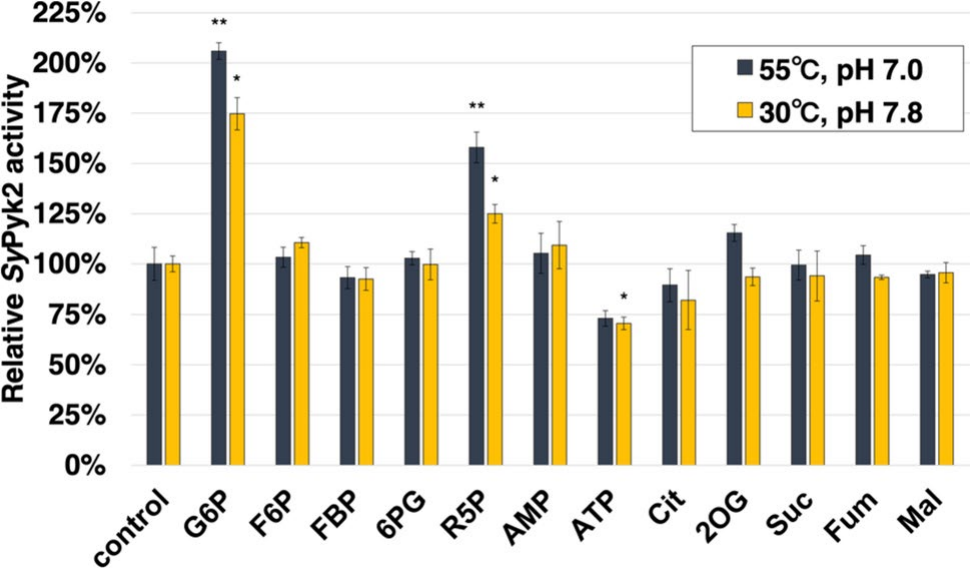
Effects of effectors for *Synechocystis* pyruvate kinase 2 (*Sy*Pyk2) activity. The effects of various metabolites on the activity *Sy*Pyk2. These experiments measured optimum conditions at 55 °C and pH 7.0 in MES-NaOH buffer (left blue bar) and intracellular conditions at 30 °C and pH 7.8 in Tris–HCl buffer (right yellow bar). The mean ± SD values were calculated from three independent experiments. The concentration of PEP and ADP were fixed at *K*_m_ (2.5 mM) and 2.0 mM, respectively. In the measurements of the saturation curves of *Sy*Pyk2, the concentrations of KCl and MgCl_2_ were 100 and 15 mM, respectively. The concentration of several effectors was 1.0 mM. G6P, glucose-6-phosphate-2Na; F6P, fructose-6-phosphate-2Na; FBP, fructose-1, 6-bisphosphate-3Na; R5P, ribose-5-phosphate-2Na; 6PG, 6-phospho-D-gluconate; AMP, adenosine monophosphate-Na; ADP, adenosine diphosphate-2Na; ATP, adenosine triphosphate-2Na; Cit, citrate-3Na; 2OG, 2-oxoglutarete; Suc, succinate-2Na; Fum: fumarate, Mal: malate-Na. The asterisks indicated significant differences between the absence and presence of the salt (Student’s *t*-test; *P < 0.01, **P < 0.005)

We measured the relative catalytic activity of *Sy*Pyk2 for PEP in the presence of sugar phosphates from the EMP/gluconeogenesis and OPP pathways at 1 mM, and PEP and ADP was fixed at *K*_m_ (2.5 mM) and 2 mM respectively, under intracellular conditions. The effectors did not affect LDH (coupled enzyme) used in the experiment. Additionally, several effector metabolites known to inhibit Pyk from the TCA cycle, such as citrate and 2OG, were added at 1 mM under optimum conditions (Wu and Turpin 1992; Knowles et al. 2001). Under optimal conditions, *Sy*Pyk2 was activated by 1 mM G6P and R5P up to 200 and 150%, respectively, and inhibited down to 75% by ATP (Fig. 6). Under intracellular conditions, *Sy*Pyk2 was activated in the presence of 1 mM G6P and R5P by 150 and 125%, respectively, and inhibited in the presence of ATP by up to 75% (Fig. 6). Unlike *Synechococcus* Pyk and green alga, *Chlamydomonas reinhardtii* Pyk, *Sy*Pyk2 was not activated by AMP or F6P and not inhibited by the TCA cycle metabolites, such as citrate, 2OG, and malate (Fig. 6; Wu and Turpin 1992; Knowles et al. 2001).

**Fig. 7:**
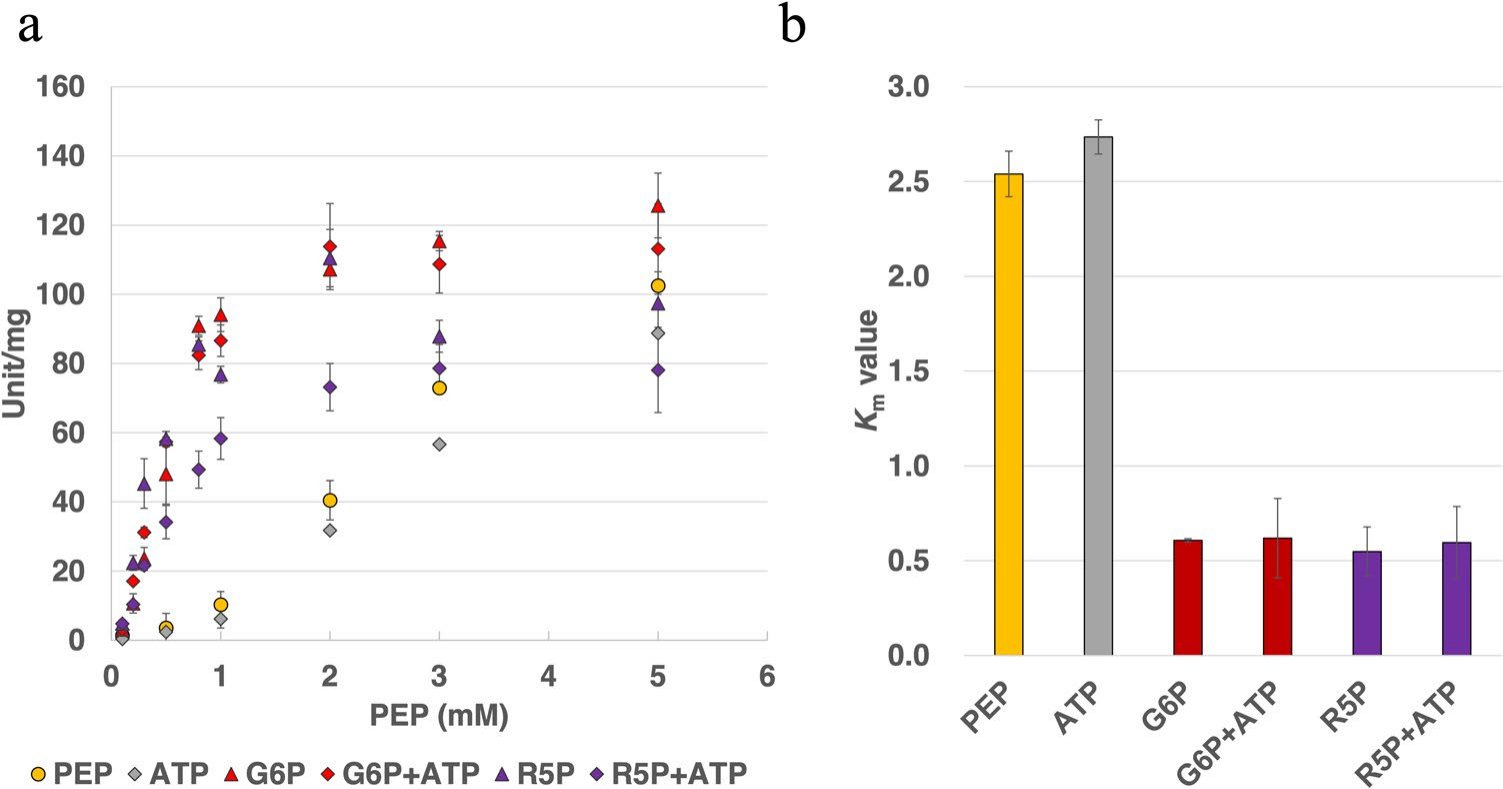
**a** Influence of several effectors for *Synechocystis* pyruvate kinase 2 (*Sy*Pyk2) activity. Circles (blue) indicated the phosphoenolpyruvate (PEP) saturation curve, squares (red and purple) indicated 1.0 mM of glucose-6-phosphate (G6P) and ribose-5-phosphate (R5P), diamonds (gray) indicated 1.0 mM of adenosine triphosphate (ATP) added, and red or purple diamonds indicated the presence of G6P and R5P with ATP added respectively. The mean ± SD values were calculated from three independent experiments. **b** This figure shows the *K*_m_ value of *Sy*Pyk2 for PEP in the presence of G6P, R5P, and ATP, coexisting intracellular conditions of 30 °C and pH 7.8 in Tris–HCl buffer. The mean ± SD values were calculated from three independent experiments

Furthermore, we calculated the kinetic parameters of *Sy*Pyk2 for PEP in the presence of activators or inhibitors under intracellular conditions. The saturation curves of *Sy*Pyk2 for PEP in the presence of G6P revealed a hyperbolic curve with *V*_max_ and *K*_m_ of 122 ± 5 unit/mg and 0.607 ± 0.01 mM, respectively, and a Hill coefficient of 2.0 ± 0.1, indicating G6P converting *Sy*Pyk2 from sigmoidal to hyperbolic kinetics (Fig. 7a and Table 1). The enzymatic activities of *Sy*Pyk2 for PEP in the presence of R5P displayed a hyperbolic curve with *V*_max_ and *K*_m_ of 125 ± 18 unit/mg and 0.548 ± 0.132 mM, respectively, and a Hill coefficient of 1.4 ± 0.2, indicating R5P altering *Sy*Pyk2 from sigmoidal to hyperbolic kinetics (Fig. 7a and Table 1). Similar to *Synechococcus* Pyk, G6P and R5P decreased the *K*_m_ value of *Sy*Pyk2 for PEP to one-fifth of its value (Knowles et al. 2001 and Table 1). Moreover, the *K*_m_ value of *Sy*Pyk2 was increased by ATP from 2.54 to 2.73 mM (Fig. 7b and Table 1). To demonstrate the effects of ATP for *Sy*Pyk2, we calculated the kinetic parameters of *Sy*Pyk2 for PEP in the presence of ATP and either G6P or R5P under intracellular conditions (Fig. 7a and b). G6P, R5P, and ATP concentrations were fixed at 1 mM. The saturation curves of *Sy*Pyk2 for PEP in the presence of ATP and G6P revealed a hyperbolic curve with *V*_max_ and *K*_m_ of 130 ± 29.5 unit/mg and 0.619 ± 0.022 mM, respectively, and a Hill coefficient of 1.73 ± 0.36 (Fig. 7a and b, and Table 1). The enzymatic activities of *Sy*Pyk2 for PEP in the presence of ATP and R5P displayed a hyperbolic curve with *V*_max_ and *K*_m_ of 84.0 ± 7.08 unit/mg and 0.572 ± 0.111 mM, respectively, and a Hill coefficient of 1.89 ± 0.41 (Fig. 7a and b and Table 1). G6P and R5P relieved the effects of ATP (Fig. 7b). Additionally, the IC_50_ (median inhibition concentration) of ATP for *Sy*Pyk2 was 4.1 mM (Supplemental Fig. 2), which was approximately 3-fold higher than that of *Synechococcus* Pyk (1.5 mM, Knowles et al. 2001). G6P and R5P increased the affinity of *Sy*Pyk2 for PEP and remained unaltered in the presence of AMP, F6P, or FBP (Figs. 6, 7a, and b, Table 1).

## Discussion

This study demonstrated the properties of *Sy*Pyk2 via biochemical analysis, with G6P and R5P increasing the affinity of *Sy*Pyk2 for PEP *in* *vitro*. The optimum pH and temperature of Pyks were discovered in bacteria (Chai et al. 2016; Kapoor and Venkitasubramanian 1983; Abbe and Yamada 1982). The optimum pH of Pyks displayed a wide peak range, from acidic to alkaline (Chai et al. 2016; Abbe and Yamada 1982). For *Synechococcus* Pyk, the optimal pH ranged from 6.0 to 7.8, and it was active in the dark (Knowles et al. 2001). The intracellular pH of *Synechocystis* under the photoautotrophic condition was 7.8 (Nakamura et al. 2021), and light to dark transition decreases the intracellular pH of other cyanobacteria from alkaline to neutral (Falkner et al. 1976; Mangan et al. 2016). Thus, the broad pH range of *Sy*Pyk2 indicated that *Sy*Pyk2 could act on PEP consumption under any cultivation conditions. The optimum temperature of *Sy*Pyk2 was 55 °C (Fig. 2c). *Synechocystis* grows at ~ 30 °C (Inoue et al. 2001), and the optimum temperature of *Sy*Pyk2 is higher than that of the cultivation conditions (Fig. 2c). Although for a short time 5 min, *Synechocystis* is viable up to 54 °C (Inoue et al. 2001). Under heat shock conditions, ATP plays a crucial role in protein maintenance through chaperones (Soini et al. 2005). The gene expression of *pyk2* increases during heat shocks (Slabas et al. 2006), and hence, *Sy*Pyk2 may contribute to ATP production by increasing its enzymatic activity.

Pyks have been studied for their properties and primary sequences (Hunsley and Suelter 1969; Cottam et al. 1969; Abdelhamid et al. 2019; 2021). All Pyks require divalent cations, such as Mg^2+^ or Mn^2+^, and numerous Pyks require K^+^ for activity (Baek and Nowak 1982; Kachmar and Boyer 1953). *Sy*Pyk2 showed Mg^2+^- and Mn^2+^-dependent activity and other bacterial Pyks, and its activity was stimulated by monovalent cations, such as K^+^, Na^+^, or NH_4_^+^(Fig. 3). Bacterial Pyks are classified into two types: PykA, which is stimulated by sugar monophosphates, such as AMP, G6P, and R5P, and PykF, activated by sugar diphosphates, such as FBP in *E*. *coli* (Kornberg and Malcovati 1973; Waygood et al. 1975, 1976). Moreover, *Sy*Pyk2 is classified as PykF (Kaneko et al. 1996). In silico analysis suggests that two isozymes, Pyk1 and Pyk2 have the same allosteric sites for G6P, R5P, FBP, AMP, and ATP (Haghighi 2021). Pyks containing Pyk1 and Pyk2 from *Synechocystis* cells are activated by G6P, F6P, R5P, and AMP but not by FBP (Knowles and Plaxton 2003). Based on these findings, we demonstrated the regulation of *Sy*Pyk2 by adding various metabolites from the OPP pathway, EMP/gluconeogenesis pathway, and TCA cycle (Fig. 7a). Our findings reveal a decrease in the *K*_m_ value of *Sy*Pyk2 with K-type characteristics, indicative of altered substrate affinity and allosteric activation by G6P and R5P (Fig. 7b and Table 1). Our results suggest that G6P and R5P may also activate *Sy*Pyk1. Therefore, *Sy*Pyk2 is dependent on divalent cations, such as Mg^2+^ and Mn^2+^, and is classified as PykA type rather than PykF, stimulated by sugar monophosphates, such as G6P and R5P, but not by AMP.

The *K*_m_ values of *Sy*Pyk2 for PEP were 40-fold higher than ADP, indicating a higher requirement for PEP in its enzymatic reaction than ADP under intracellular conditions (Table 1). The *K*_m_ value of *Synechococcus* Pyk for PEP is higher than that for ADP (Knowles et al. 2001). The *K*_m_ value of *Sy*Pyk2 for PEP is higher than *Synechococcus* Pyk 5-fold (Table 1 and Knowles et al. 2001). Additionally, the absolute concentration of ADP in *Synechocystis* cells is three times higher than that of PEP (Dempo et al. 2014). These data indicate that these two Pyk enzymes, *Synechocystis* and *Synechococcus*, are limited by PEP concentration in their reactions under photosynthetic conditions. However, its mechanism is different. *Synechococcus* Pyk has high PEP affinity and allosteric inhibition by citrate and ATP (Knowles et al. 2001). *Synechocystis* has a low affinity and not inhibited by either citrate or ATP (Table 1 and Fig. 6). Thus, these findings suggested that the availability of PEP limited the enzymatic activity of *Sy*Pyk2 for the flux of PEP to pyruvate via *Sy*Pyk2 under photosynthetic conditions.

Rapid glycogen catabolism induces glucan polymer such as G6P and signaling metabolites such as 2OG occur during nitrogen depletion (Joseph et al. 2014), indicating that G6P may activate *Sy*Pyk2. Although *Synechococcus* Pyk is repressed by 2OG, *Sy*Pyk2 is not (Fig. 6). However, a previous study reveals that *pyk1* expression is induced 3.5-fold, and *pyk2* expression is reduced by half during nitrogen-deficient conditions (Osanai et al. 2006). Hence, these findings indicate that to provide a carbon source to the TCA cycle for 2OG production, *Sy*Pyk2 may act during the initial stages of nitrogen depletion through G6P and R5P activation and then be replaced with *Sy*Pyk1. Furthermore, *pyk1* is regulated by several nitrogen-related regulators such as SigE, Rre37 thorough with NtcA (Giner-Lamia et al. 2017; Iijima et al. 2014; Osanai et al. 2005). Therefore, in *Synechocystis*, we consider *Sy*Pyk1 and *Sy*Pyk2 to mainly function as pyruvate kinase during the late and initial nitrogen depletion stages, respectively.

In comparison to the *K*_m_ value of PEP for *Synechocystis* PEPC (0.34 mM: Takeya et al. 2017; 0.88 mM: Scholl et al. 2020 and Table 2), *Sy*Pyk2 (2.54 mM, Table 1) required more than 2-fold higher concentration of PEP (Tables 1 and 2). The *K*_m_ value of *Sy*Pyk2 for PEP was decreased from 2.54 to 0.607 or 0.548 mM by G6P or R5P, respectively (Fig. 7a and b and Table 1). The higher PEP requirement and the enhanced affinity of *Sy*Pyk2 for PEP by G6P and R5P, suggesting a role for *Sy*Pyk2 in *Synechocystis* cells. In a previous study, Pyk from *Synechocystis* demonstrated a higher Pyk activity under heterotrophic conditions than under photoautotrophic and mixotrophic conditions (Knowles and Plaxton 2003). Recently, ME, which generates pyruvate from malate by the ME-dependent TCA cycle, was reportedly active under photoautotrophic conditions (Katayama et al. 2022), indicating that pyruvate is synthesized by ME and not by Pyk (Bricker et al. 2004; Qian et al. 2018). The pathway involving PEPC, MDH, and ME constitutes an alternate route for pyruvate formation in *Synechocystis* cells under photosynthetic conditions (You et al. 2014; Bricker et al. 2004). ATP functions as an inhibitor of *Synechococcus* Pyk, which is the homolog of *Sy*Pyk2 (Knowles et al. 2001). This observation suggests that the lack of Pyk flux under photosynthetic conditions can be attributed to ATP inhibition (Bricker et al. 2004). In *E*. *coli*, the intracellular concentration of ATP is suggested to be 0.6 mM (Boecker et al. 2019) and not much different from *Synechocystis* (Wan et al. 2017). G6P and ATP exhibit comparable concentrations, approximately 1.84*10^0^ and 2.14*10^0^ µmol/g-dry cell weight, respectively (Dempo et al. 2014). R5P is one-tenth of the concentration of ATP, amounting to 1.95*10^–1^ µmol/g-dry cell (Dempo et al. 2014). Hence, to demonstrate the in vivo effects of metabolites, we maintained uniform effector concentrations at 1 mM. Inhibition by 1 mM ATP decreased the *V*_max_ and increased the *K*_m_ value of *Sy*Pyk2 activity (Table 1 and Fig. 7b). The *V*_max_ of *Sy*Pyk2 for PEP decreased from 119 ± 7 to 107 ± 5, and the *K*_m_ value of *Sy*Pyk2 for PEP increased from 2.54 to 2.74 mM (1.07-fold) (Fig. 7b and Table 1). In comparison, the *K*_m_ value of *Synechococcus* Pyk increased from 0.54 to 0.75 mM (1.37-fold) by 0.5 mM ATP (Knowles et al. 2001). Furthermore, compared to the IC_50_ of *Synechococcus* Pyk for ATP (1.5 mM: Knowles et al. 2001), *Sy*Pyk2 (4.1 mM: Supplemental Fig. 2) was approximately 3-fold higher, indicating that *Sy*Pyk2 is less affected by ATP than *Synechococcus* Pyk. Additionally, the presence of G6P and R5P alleviated the inhibitory effects of ATP, reducing the *K*_m_ value of the substrate concentration to approximately one-fifth (Fig. 7a and b). Our results showed that the effects of ATP on *Sy*Pyk2 are less potent than those of G6P and R5P. Considering the IC_50_ and the slight increase in the *K*_m_ value by ATP, it suggests that *Sy*Pyk2 is less influenced by ATP. Therefore, we conclude that the low flux of PEP to pyruvate via Pyk is due to its extremely low affinity for PEP (Tables 1 and 2; Knowles et al. 2001) and the absence of activators such as G6P and R5P under photosynthetic conditions.

**Fig. 8:**
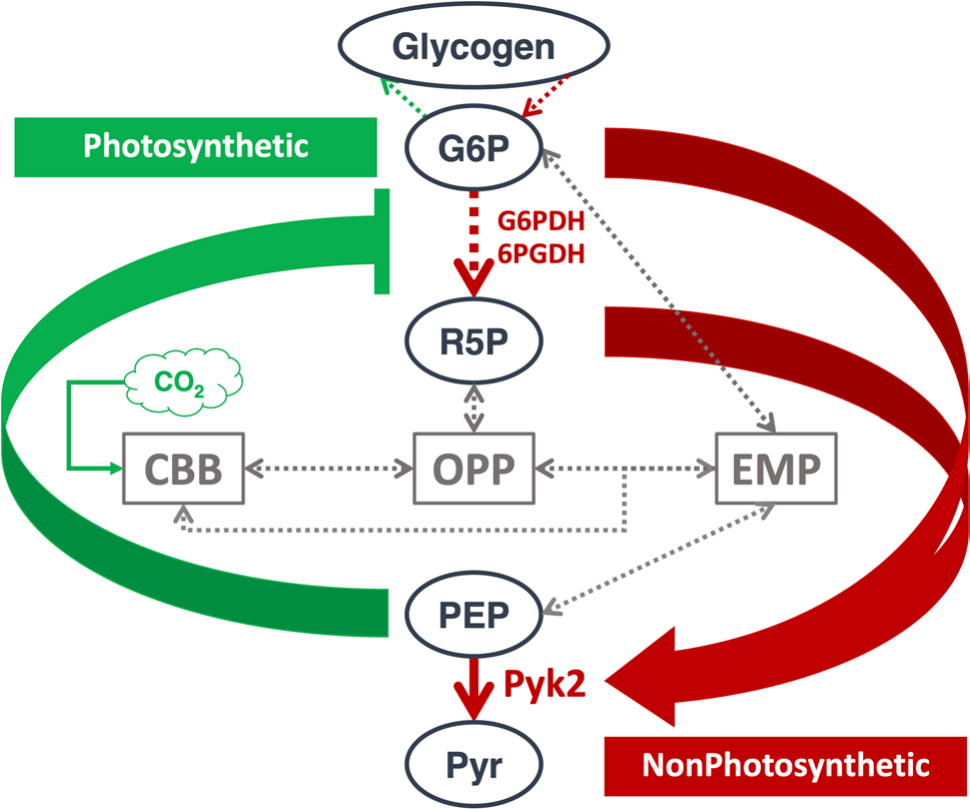
Model of glucose-6-phosphate (G6P), ribose-5-phosphate (R5P) and phosphoenolpyruvate (PEP) regulation of carbon flow under photosynthetic or nonphotosynthetic conditions in *Synechocystis*sp. PCC 6803. EMP, Embden–Meyerhof–Parnas pathway; OPP, oxidative pentose phosphate pathway; CBB, Calvin Benson Bassham cycle; Pyr, pyruvate; G6PDH, glucose-6-phosphate dehydrogenase; 6PGDH, 6-phosphogluconate dehydrogenase; Pyk2, pyruvate kinase 2

The flux through the OPP pathway increases under nonphotosynthetic conditions (Wan et al. 2017) relative to photosynthetic conditions (Young et al. 2011), indicating a significant elevation in the levels of G6P and R5P under nonphotosynthetic conditions. Moreover, PEP increases and decreases under photosynthetic and nonphotosynthetic conditions, respectively (Werner et al. 2019). Consequently, the accumulation of G6P and R5P under nonphotosynthetic conditions upregulates the *Sy*Pyk2 reaction. Following *Sy*Pyk2 activation in the presence of G6P and R5P, PEP is consumed by *Sy*Pyk2, alleviating glucose-6-phosphate dehydrogenase (G6PDH encoded by *zwf*, slr1843) inhibition, a rate-limiting enzyme of the OPP pathway (Ito and Osanai 2020). Furthermore, PEP consumption via *Sy*Pyk2 activating relieving the inhibition of 6-phosphogluconate dehydrogenase (6PGDH encoded by *gnd*, sll0329) (Ito and Osanai, 2018), an enzyme involved in the third reaction of the OPP pathway (Fig. 8). In this feedforward regulation, *Sy*Pyk2 primarily acts as relieving the sugar catabolic repression under nonphotosynthetic conditions.

This study offers valuable insights into the biosynthesis and fermentation of metabolites associated with pyruvate metabolism, particularly PEP consumption in *Synechocystis*.

This study demonstrated that the regulation of *Sy*Pyk2 is dependent on PEP accumulation, the presence of G6P, R5P, and divalent cations, such as Mg^2+^ and Mn^2+^, rather than pH and ATP. Therefore, our experiments indicated that *Sy*Pyk2 contributed less to PEP consumption under photosynthetic conditions and that it plays a pivotal role in sugar catabolism under nonphotosynthetic conditions in response to sugar phosphate accumulation.

### Supplementary Information

Below is the link to the electronic supplementary material.

Supplementary file1 (TIFF 35159 KB)

Supplementary file2 (TIFF 35159 KB)

Supplementary file3 (DOCX 17 KB)

## Data Availability

Not applicable.
