# Supplementary figures and images for: Pyruvate kinase 2 from Synechocystis sp. PCC 6803 increased substrate affinity via glucose-6-phosphate and ribose-5-phosphate for phosphoenolpyruvate consumption
Source: Plant Mol Biol. 2024 May 17;114(3):60. doi: 10.1007/s11103-023-01401-0 (PMC11101554; doi:10.1007/s11103-023-01401-0)

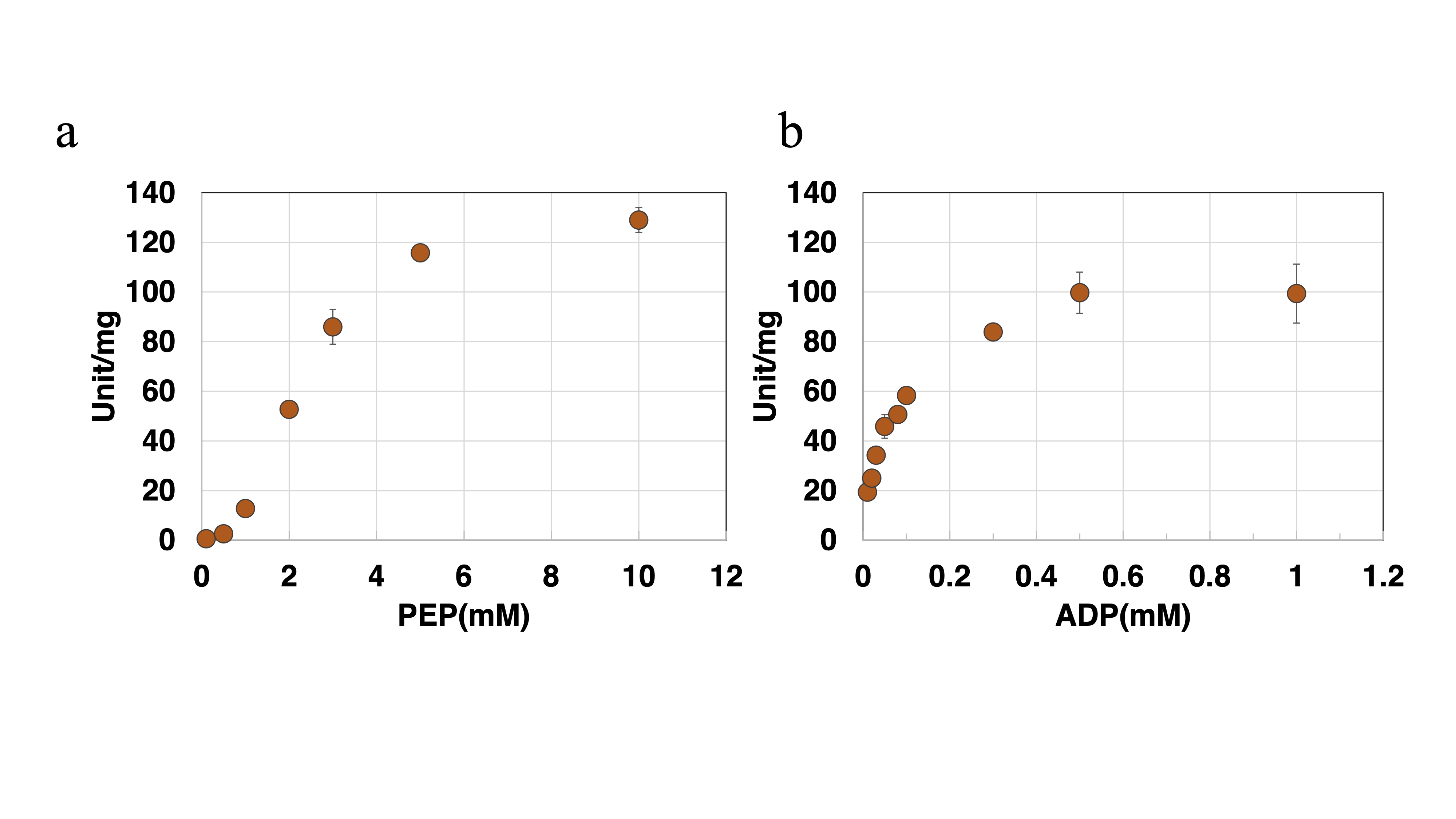

Supplement: Supplementary file 1 — Supplementary file1 (TIFF 35159 KB) [file 11103_2023_1401_MOESM1_ESM.tiff]

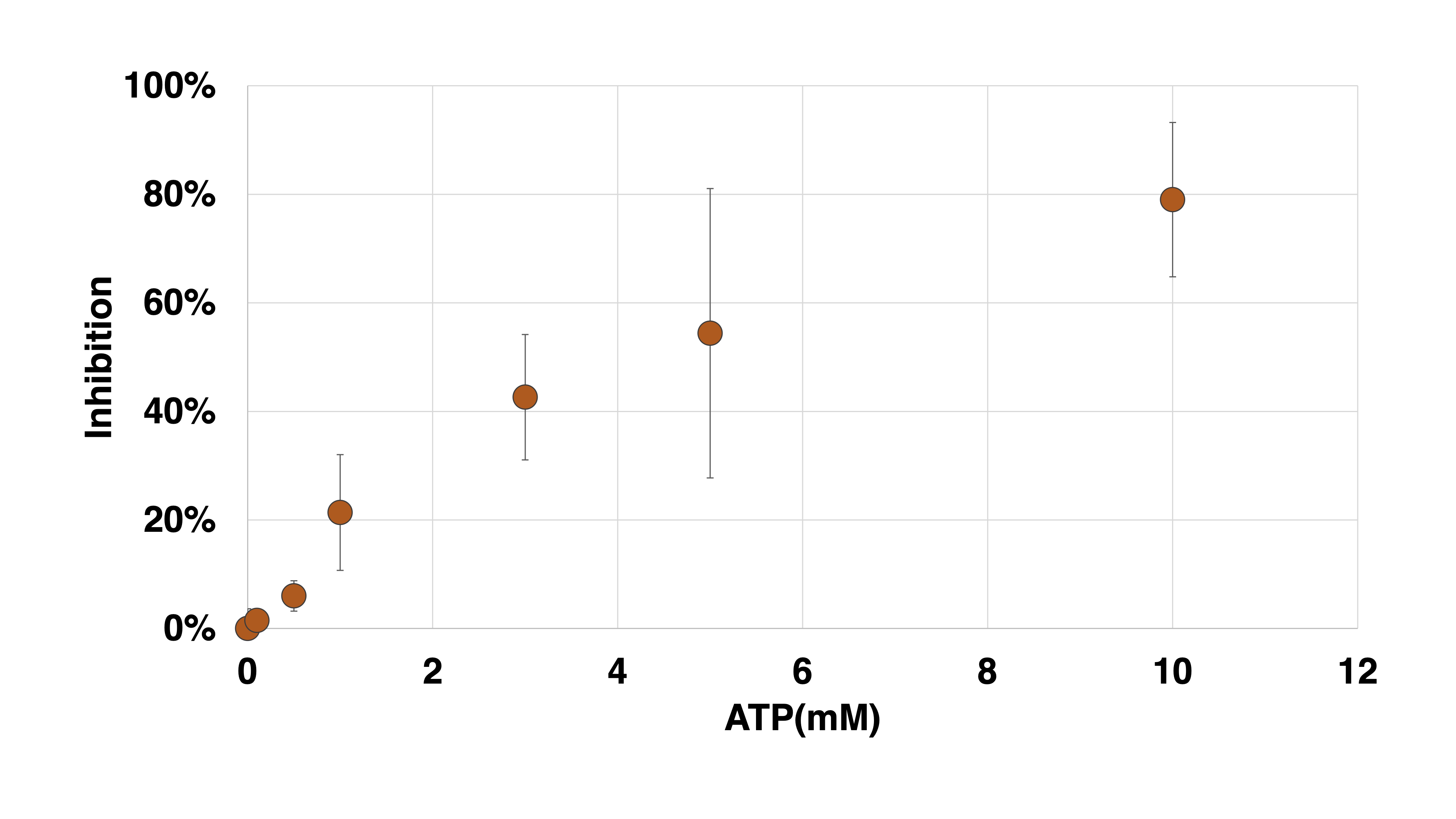

Supplement: Supplementary file 2 — Supplementary file2 (TIFF 35159 KB) [file 11103_2023_1401_MOESM2_ESM.tiff]
