# Supplementary material for: Pyruvate kinase 2 from Synechocystis sp. PCC 6803 increased substrate affinity via glucose-6-phosphate and ribose-5-phosphate for phosphoenolpyruvate consumption
Source: Plant Mol Biol. 2024 May 17;114(3):60. doi: 10.1007/s11103-023-01401-0 (PMC11101554; doi:10.1007/s11103-023-01401-0)
Supplement: Supplementary file 3 — Supplementary file3 (DOCX 17 KB) [file 11103_2023_1401_MOESM3_ESM.docx]

**Supplementary figure legends**

**Supplemental Fig. 1** Saturation curves of *Synechocystis* sp. PCC 6803 pyruvate kinase 2 (*Sy*Pyk2) for phosphoenolpyruvate (PEP) and adenosine diphosphate (ADP). (a) Saturation curves of *Sy*Pyk2 for PEP. These measurements were performed in optimum *Synechocystis* phosphoenolpyruvate carboxylase (PEPC) conditions at 30°C and pH 7.0 in Tris-HCl (Takeya et al. 2017). The adenosine diphosphate (ADP) concentration was 2.0 mM. The concentrations of KCl and MgCl_2_ were 100 and 15 mM, respectively. Mean ± SD values were calculated from three independent experiments. (b) Saturation curves of *Sy*Pyk2 for ADP. These measurements were performed in optimum *Synechocystis* PEPC conditions at 30°C and pH 7.0 in Tris-HCl (Takeya et al. 2017). The PEP concentration was 5 mM. The concentrations of KCl and MgCl_2_ were 100 and 15 mM, respectively. Mean ± SD values were calculated from three independent experiments.

**Supplemental Fig. 2** Adenosine triphosphate (ATP) inhibition of *Sy*Pyk2. The orange circle indicated inhibited *Sy*Pyk2 activity. This experiment measured intracellular conditions at 30°C and pH 7.8 in Tris-HCl buffer. The concentration of phosphoenolpyruvate (PEP) and adenosine diphosphate (ADP) was 5 and 2 mM. The concentrations of KCl and MgCl_2_ were 100 and 15 mM, respectively. Mean ± SD values were calculated from three independent experiments. Half-maximal (50%) inhibitory concentration (IC_50_) was 4.1 mM of ATP.
